# Comparative effects of electronic and traditional cigarette use on pulmonary function, autonomic regulation, and exercise capacity in young adults

**DOI:** 10.1007/s11845-025-04271-1

**Published:** 2026-01-15

**Authors:** Nurel Erturk, Hayrul Nisa Karababa

**Affiliations:** Present Address: Tarsus University, Faculty of Health Sciences, Department of Physiotherapy and Rehabilitation, Mersin, Türkiye

**Keywords:** E-cigarette vapor, Smoke, Lung function, Heart rate variability

## Abstract

**Background:**

E-cigarette use among university students has been increasing. While substantial information exists on the impact of e-cigarette use on respiratory function, limited data exists on its early effects on cardiac autonomic function and exercise capacity in young individuals.

**Aims:**

This study evaluated respiratory function, heart rate variability (HRV), and exercise capacity in electronic cigarette users compared to traditional cigarette smokers and non-smokers among university students.

**Methods:**

Sixty-one healthy volunteers aged 18-35 years were divided into three groups: nonsmokers (n=20), tobacco smokers (n=21), and e-cigarette users (n=20). Respiratory function was assessed using spirometry, HRV was measured using a Polar H10 chest strap, and exercise capacity was evaluated using the incremental shuttle walk test (ISWT).

**Results:**

Results showed forced vital capacity was significantly higher in non-smokers than in traditional cigarette and e-cigarette users (P =0.013). No significant differences were found in spirometry parameters, HRV indices, total shuttle number, or ISWT distance. The percentage of predicted distance was significantly higher in non-smokers than other groups (p=0.018). The percentage of maximal heart rate and post-test heart rate differed significantly between groups (p = 0.004; η² = 0.172 and η² = 0.174), with traditional cigarette smokers showing lower values than non-smokers and e-cigarette users. Post-exercise dyspnea scores were significantly lower in e-cigarette users than nonsmokers (p=0.009).

**Conclusion:**

Both tobacco and e-cigarette use may negatively affect respiratory function and exercise performance of university students. Further research is needed to clarify the long-term health effects of electronic cigarette use in both healthy and chronic disease populations.

## Introduction

Currently, cigarette smoking is one of the most significant health problems. Tobacco addiction, which causes the death of more than 8.5 million people worldwide each year, is a preventable public health issue on a global scale. The presentation of e-cigarettes, known as electronic nicotine delivery systems, is less harmful than traditional cigarettes, and claims that they reduce cigarette use and addiction have led to the widespread adoption of this product worldwide [1]. Electronic cigarettes (e-cigarettes) resemble cigarettes in appearance and provide a cigarette-like pleasures during use. While the rate of e-cigarette use was 4.5% in 2011, it had increased to 13.4% in 2014. E-cigarettes are introduced into the market in various designs and types each day, and their demand is rapidly increasing [2]. Today, e-cigarettes, which are legally sold in many countries around the world, have become the choice of many users for reasons such as being preferred primarily as an alternative to those who want to quit smoking, offering a variety of flavors, being more affordable, being considered healthier than tobacco, and out of curiosity.

According to previous research, e-cigarette users are generally young adults [3]. E-cigarettes operate using electrical power to generate heat, which in turn vaporizes the e-cigarette liquid. Unlike regular cigarette smoke, electronic cigarette vapors do not contain only carbon monoxide. In general, e-cigarette liquids contain nicotine and propylene glycol (PG). Owing to the battery-powered system, micron-sized particles are sprayed and reach the lungs when inhaled. Over time, the particles delivered to the airways can cause lung infections. Therefore, there are concerns regarding whether they pose a risk to lung health. However, research on the health benefits of e-cigarettes remains insufficient. However, there are conflicting results regarding their effects on the respiratory system [1, 4]. Among those who use only e-cigarettes, negative respiratory symptoms such as increased cough, dry mouth/mouth irritation, phlegm, wheezing, shortness of breath, chest pain, and palpitations have been reported [5].

Cigarette consumption is associated with serious cardiovascular diseases. The formation of atherosclerotic plaques affects coronary arteries. Smoking leads to endothelial damage, reduced oxygen utilization by the pericardium, and decreased capacity of the muscles to carry oxygen during exercise. It has been reported that individuals who smoke experience reduced respiratory functions, decreased exercise capacity, and impaired chronotropic responses [6]. Heart rate variability (HRV), which is a measure of cardiac vagal control, is positively associated with healthy cardiac function and longevity. Conversely, low HRV predicts an increased risk of all-cause mortality and death following fatal myocardial infarction [7]. Several observational studies have reported the effects of chronic smoking on HRV in heavy smokers [8]. Evidence from laboratory and observational studies has shown that both acute and chronic smoking reduce HRV [8].

The literature presents conflicting data on the impact of e-cigarettes on pulmonary health. To the best of our knowledge, no study has been conducted that compared respiratory function, heart rate variability, and exercise capacity among e-cigarette users, traditional cigarette smokers, and non-smokers. Given the widespread use of e-cigarettes, particularly due to the minimum age restrictions and their aromatic appeal, it is imperative to investigate the initial cardiac and respiratory effects of e-cigarette consumption among university students. This study aimed to assess respiratory function, exercise capacity, and heart rate variability in university students who use e-cigarettes and compare these parameters with those of traditional cigarette users and non-smokers.

## Methods

### Study design and participants

University students aged 18–30 who use e-cigarettes, traditional cigarettes, or did not smoke (never smoked) were included in this cross-sectional study. Individuals who had previously smoked but had quit smoking, those who used both e-cigarettes and traditional cigarettes, those diagnosed with chronic lung and heart diseases, and those with orthopedic or neurological conditions that would prevent them from performing the exercise test were excluded from the study. Descriptive information of the individuals included in the study, especially the amount and frequency of cigarette use, was collected using a data registration form. In addition, participants’ respiratory function, exercise capacity, and heart rate variability were assessed. Data from three groups–e-cigarette users, traditional cigarette users, and non-smokers (never smoked)–were compared. This study was approved by the Tarsus University Non-Interventional Ethics Committee (No. 2025/69).

G*Power 3.0.18 (Heinrich-Heine-Universität Düsseldorf, Germany) was used to determine the sample size. Based on the study by Polosa et al., to detect a significant difference in the FEV1/FVC ratio between the study groups, an effect size (d) of 1.19, 95% power (1-β = 0.95), and alpha level (type I error) of 0.05, were used to detect a sample size of 20 per group between the total of 60 participants [1].

#### Demographic information

Age, sex, height (m), weight (kg), body mass index (kg/m2), type of cigarette, and cigarette exposure (packs/year) were recorded.

#### Pulmonary function

The pulmonary function test was performed in a sitting position using a spirometer device in accordance with the criteria of the American Thoracic Society and European Respiratory Society. Forced vital capacity (FVC) is the volume of air exhaled forcefully and deeply after a deep inspiration; the forced expiratory volume in the first second (FEV1) is the volume of air exhaled in the first second of the forced vital capacity maneuver; the FEV1/FVC ratio, peak expiratory flow (PEF), and forced expiratory flow at 25–75% of exhalation (FEF 25–75%) were measured. The highest value among the three consistent measurements was recorded, and the measurements were compared with the expected values determined based on the patients’ age, sex, body weight, and height and expressed as a percentage [9].

#### Evaluation of exercise capacity

Exercise capacity was assessed using the incremental shuttle walking test (ISWT). The covered distance was calculated as the number of completed shuttles (10 m). The lowest saturation and the highest heart rates were recorded during the test. Heart rate, blood pressure, oxygen saturation, perceived dyspnea, and fatigue were recorded before and after the test [10]. In the evaluation of the ISWT distance results, the expected values were determined using the equations $ISWT=1449.701$ - $\lbrack11.735\times\;age\rbrack$ + $\lbrack241.897\rbrack\;--\;\lbrack5.686\times BMI\rbrack$ for men (where BMI is the value determined according to body mass index), $ISWT=1449,701$ - $\lbrack11,735\times\;age\rbrack$ - $\lbrack5.686\times BMI\rbrack$ and for women.

**Heart rate variability (HRV)** was assessed using a Polar H10 chest strap. The Polar H10 device is an HRV sensor with a gold standard for high sensitivity and accuracy, and it uses a wearable chest strap. The electrical activity generated by the heartbeat is transmitted wirelessly from the sensor on the chest strap to a suitable receiver (smart watch, mobile device, or other similar receivers) [11]. Heart rate variability (HRV) parameters were calculated using Kubios HRV software (Kubios Oy, Kuopio, Finland) based on successive normal-to-normal (NN) R–R intervals. The validity and reliability of the Polar H10 chest strap for short-term HRV assessment at rest have been demonstrated previously, with intraclass correlation coefficients (ICC) ranging from 0.85 to 0.95 in healthy women and men [12]. The participants were assessed in a seated position for 5 min [13]. The calculated time-domain HRV parameters were the mean RR interval (MeanRR), standard deviation of NN intervals (SDNN), root mean square of successive differences (RMSSD), parasympathetic nervous system (PNS) index, and heart rate. Respiratory rate was analyzed as an independent physiological variable. HRV parameters were interpreted based on commonly reported reference ranges for short-term resting recordings in healthy adults, with lower RMSSD and SDNN values indicating reduced autonomic modulation [14].

### Statistical analysis

Statistical analyses were performed using IBM SPSS Statistics for Windows version 22. The suitability of the variables for normal distribution was examined using visual (histogram and probability plots) and analytical methods (Kolmogorov-Smirnov/Shapiro-Wilk tests). Demographic data and results of the evaluated parameters were assessed using descriptive analyses. When the parameters showed a normal distribution, the results were expressed as mean (M) ± standard deviation (SD). The results are expressed as median (interquartile range) when the parameters did not show a normal distribution. For normally distributed data, the Independent Samples T-Test was used for binary comparisons; for non-normally distributed data, the Mann-Whitney U test was used. Analysis of variance was used to compare quantitative data among the three normally distributed groups, analysis of variance (ANOVA) was used. When a significant main effect was detected, post hoc pairwise comparisons were performed using Tukey’s honestly significant difference (HSD) test. Effect sizes were calculated using eta squared (η²) to quantify the magnitude of between-group differences.

## Results

A total of 61 volunteers participated in the study. Participants were 20 non-smokers, 21 traditional cigarette users, and 20 electronic cigarette users. The demographic characteristics of the study groups are shown in Table 1. The proportion of women in the non-smoking group was 95%, whereas it was 71.4% among traditional cigarette users, and 75% among electronic cigarette users. The proportion of men was 5%, 28.6%, and 25%, respectively. Sex distribution did not differ significantly between groups (Fisher–Freeman–Halton exact test, *p* = 0.122). No significant differences were observed between the groups in terms of age (*p* = 0.607) or BMI (*p* = 0.424). The average cigarette exposure in the traditional cigarette user group was 0.75 ± 0.59 packs, with an average usage duration of 55.35 ± 32.31 months. The usage duration for electronic cigarette users was 29.35 ± 19.63 months.

**Table 1:** Demographic information of the groups

	Non-smokers (*n* = 20)	Traditional cigarette smokers (*n* = 21)	E-cigarette users (*n* = 20)	*p*
Gender, female, n(%)	19 (95.0)	15 (71.4)	15 (75.0)	0.122
Age, years	21.40 ± 1.50	21.09 ± 1.64	20.20 ± 5.77	0.607
BMI, kg/m^2^	22.20 ± 3.85	21.47 ± 3.20	22.96 ± 3.77	0.424
Exposure to cigarettes (paket)	0.0 ± 0.0	0.75 ± 0.59	0.0 ± 0.0	n/a
Exposure to smoking (month)	0.0 ± 0.0	55.35 ± 32.31	29.35 ± 19.63	n/a

No significant differences were observed between the groups in any of the HRV parameters (Table 2). The time-domain parameters, RMSSD and SDNN, remained at the lower limit of normal adult values across all groups. The PNS index values were negative in all groups, with no significant difference. Respiratory rate values were also similar between the groups and remained within the normal adult range ( 15–16 breaths/min).

**Table 2:** Comparison of heart rate variability and respiratory function test results between groups

	Non-smokers (*n* = 20)	Traditional cigarette smokers (*n* = 21)	E-cigarette users (*n* = 20)	Effect size (Ƞ^2^)	*p*
Heart rate, bpm	80.05 ± 7.93	78.19 ± 9.33	83.45 ± 13.69	0.043	0.282^a^
RMSSD, ms	44.40 ± 23.28	41.61 ± 19.09	39.70 ± 27.08	0.007	0.815^a^
PNS index, a.u.	-5.70 ± 22.68	-0.59 ± 0.99	-0.91 ± 1.41	0.033	0.448^a^
MeanRR, ms	757.21 ± 80.47	733.82 ± 192.51	740.60 ± 141.73	0.005	0.877^a^
SDNN, ms	46.13 ± 18.44	79.98 ± 154.02	45.08 ± 22.79	0.032	0.388^a^
Respiratory rate, breaths/min	15.83 ± 3.52	16.41 ± 4.39	15.61 ± 3.63	0.035	0.792^a^
FVC, L	3.49 ± 0.69	4.17 ± 0.89	4.22 ± 0.92	**0.141**	**0.013** ^**a**^
					**0.035** ^**b**^
					**0.023** ^**d**^
FEV_1_, L	2.88 ± 0.68	3.26 ± 0.68	3.23 ± 0.82	0.056	0.194^a^
FEV_1_/FVC, %	80.95 ± 12.98	79.25 ± 11.82	77.25 ± 13.84	0.014	0.665^a^
PEF, L/min	266.15 ± 86.14	270.06 ± 114.24	266.60 ± 120.18	0.000	0.992^a^
FEV_25 − 75_, L/s	2.99 ± 0.92	14.92 ± 52.27	3.18 ± 1.06	0.035	0.520^a^

RMSSD: Root mean square of successive differences, PNS: Parasympathetic nervous system, MeanRR: Mean of all normal to normal RR intervals, SDNN: Standard deviation of all normal-to-normal RR intervals, FVC: Forced vital capacity, FEV₁: Forced expiratory volume in one second, FEV₁/FVC: Ratio of forced expiratory volume in one second to forced vital capacity, PEF: Peak expiratory flow, FEF₂₅-₇₅: Forced expiratory flow at 25–75% of FVC, a.u.: arbitrary units

^a^ Comparison of the non-smoking, regular cigarette smoking, and electronic cigarette smoking groups

^b^ Comparison of the non-smoking and regular cigarette smoking groups

^c^ Comparison of the electronic cigarette smoking and regular cigarette smoking groups

^d^ Comparison of the electronic cigarette smoking and non-smoking groups

According to the results of the pulmonary function tests, a significant difference was found between the groups only in the FVC values (*p* = 0.013). Subgroup analyses showed that this difference was significant between non-smokers and both traditional (*p* = 0.035) and electronic cigarette users (*p* = 0.023). No statistically significant differences were found between the groups for the other parameters, namely FEV₁ (*p* = 0.194), FEV₁/FVC (*p* = 0.665), PEF (*p* = 0.992), and FEV₂₅₇₅ (*p* = 0.520) (Table 2).

According to the shuttle walking test results, a significant difference was detected between the groups in terms of the ISWT distance percentage (*p* = 0.018 and *p* = 0.043, respectively). However, no significant differences were observed in the total number of shuttles (*p* = 0.323) or distance (*p* = 0.240).

The percentage of the maximum heart rate achieved during exercise also showed a significant difference between the groups (*p* = 0.004). In subgroup analyses, this difference was found to be between non-smokers and traditional cigarette users (*p* = 0.006), and between electronic cigarette users and traditional cigarette users (*p* = 0.024). Similarly, the post-test heart rate was significantly different between the groups (*p* = 0.004), with differences observed between non-smokers and traditional cigarette users (*p* = 0.006), and between electronic cigarette and traditional cigarette users (*p* = 0.019) (Table 3).

**Table 3:** Comparison of the groups’ increasing speed shuttle walk test results

	Non-smokers (*n* = 20)	Traditional cigarette smokers (*n* = 21)	E-cigarette users (*n* = 20)	Effect size (Ƞ^2^)	*p*
Shuttle number	83.75 ± 12.40	76.57 ± 20.27	77.90 ± 22.22	0.028	0.323^a^
Distance (m)	836.0 ± 122.44	765.71 ± 202.79	745.50 ± 269.39	0.035	0.240^a^
ISWT distance, % predicted	0.77 ± 0.10	0.66 ± 0.14	0.65 ± 0.25	**0.094**	**0.018** ^**a**^
					**0.043** ^**b**^
HR _max_, % predicted	86.16 ± 9.33	74.93 ± 13.97	84.34 ± 9.33	**0.172**	**0.004** ^**a**^
					**0.006** ^**b**^
					**0.024** ^**c**^
HR Baseline, bpm	83.35 ± 12.51	88.66 ± 12.92	88.08 ± 13.89	0.038	0.330^a^
HR post-test, bpm	171.10 ± 18.43	149.04 ± 27.90	168.40 ± 18.19	**0.174**	**0.004** ^**a**^
					**0.006** ^**b**^
					**0.019** ^**c**^
HR 1.mn recovery, bpm	133.65 ± 14.10	130.52 ± 44.44	124.70 ± 20.99	0.016	0.631^a^
HR 3.mn recovery, bpm	111.95 ± 7.94	108.66 ± 12.17	154.60 ± 199.71	0.034	0.371^a^
HR 5.mn recovery, bpm	105.70 ± 8.53	103.33 ± 11.04	103.95 ± 10.73	0.010	0.745^a^
SPO_2_ Baseline, %	97.75 ± 2.07	97.66 ± 1.90	96.70 ± 5.19	0.020	0.554^a^
SPO_2_ post-test, %	98.10 ± 1.07	97.33 ± 2.15	97.15 ± 2.10	0.049	0.234^a^
SPO_2_ 1.mn recovery, %	98.45 ± 0.88	96.28 ± 7.68	97.90 ± 1.20	0.041	0.298^a^
SPO_2_ 3.mn recovery, %	98.10 ± 0.96	97.04 ± 14.81	97.95 ± 1.14	0.005	0.862^a^
SPO_2_ 5.mn recovery, %	97.65 ± 1.03	97.95 ± 1.07	98.00 ± 1.21	0.020	0.559^a^
Dyspnea Baseline	0.95 ± 1.50	1.71 ± 1.45	1.00 ± 1.12	0.065	0.144^a^
Dyspnea post-test	8.80 ± 1.54	8.33 ± 1.52	7.05 ± 2.23	**0.150**	**0.009** ^**a**^
					**0.009** ^**d**^
Dyspnea 1. mn recovery	6.15 ± 2.00	5.85 ± 1.59	4.90 ± 2.07	0.076	0.102^a^
Dyspnea 3. mn recovery	3.15 ± 1.89	3.19 ± 1.47	2.60 ± 2.03	0.023	0.516^a^
Dyspnea 5. mn recovery	1.25 ± 1.48	1.66 ± 1.23	1.10 ± 1.44	0.031	0.405^a^
Perception of fatigue Baseline	2.05 ± 2.50	2.76 ± 1.60	2.85 ± 1.87	0.032	0.394^a^
Perception of fatigue post-test	7.25 ± 2.57	6.95 ± 1.82	7.25 ± 1.91	0.005	0.874^a^
Perception of fatigue 1. mn recovery	5.45 ± 2.35	5.57 ± 1.32	5.00 ± 2.15	0.016	0.629^a^
Perception of fatigue 3. mn recovery	3.55 ± 2.21	3.80 ± 1.20	3.25 ± 2.19	0.015	0.649^a^
Perception of fatigue 5. mn recovery	1.80 ± 1.76	2.52 ± 1.24	2.05 ± 2.06	0.031	0.396^a^

ISWT, mental shuttle walk test; HR, heart rate; HR _max_: maximum heart rate; SpO_2_, oxygen saturation

^a^ Comparison of non-smoking, traditional cigarette smoking, and electronic cigarette smoking groups

^b^ Comparison of the non-smoking and traditional cigarette smoking groups

^c^ Comparison of the electronic cigarette smoking and traditional cigarette smoking groups

^d^ Comparison of the electronic cigarette smoking and non-smoking groups

The dyspnea scores at the end of exercise were significantly different between the groups (*p* = 0.009). This difference was found to result from a comparison between e-cigarette users and nonsmokers (*p* = 0.009). No significant differences were found between non-smokers and traditional cigarette users (*p* = 0.685). No significant differences were found in the other dyspnea and fatigue parameters (*p* > 0.05) (Table 3).

## Di̇scussi̇on

The main findings indicated that nonsmokers had significantly higher FVC values than cigarette and e-cigarette users, reflecting better pulmonary function. Non-smokers also showed superior exercise capacity and higher maximum heart rate during testing. Although heart rate variability parameters did not differ between the groups, e-cigarette users reported higher dyspnea scores during exercise. Additionally, all groups showed delayed post-test heart rate recovery, suggesting impaired autonomic regulation. These findings indicate that both traditional cigarette and e-cigarette use may negatively affect cardiopulmonary function in young adults.

The FVC values of non-smokers were significantly higher than those of tobacco and e-cigarette users. This result is consistent with literature demonstrating the negative effects of smoking on lung function. The effect size analysis showed that FVC demonstrated a moderate group effect (η² = 0.141) with statistically significant post-hoc differences, suggesting that FVC was the only respiratory parameter with a potentially clinically meaningful difference between the groups. Traditional cigarette smoke reduces lung elasticity and decreases FVC values by causing inflammation in the airways, increasing mucus production, and damaging alveolar structures through toxic gases and particles [15]. Similarly, although e-cigarettes are marketed as less harmful, the inhalation of nicotine, propylene glycol, glycerol, and metal particles in aerosols can trigger oxidative stress and inflammation, negatively affecting respiratory function [16].

When evaluated in terms of exercise performance, the non-smoking group had a higher exercise capacity and achieved maximum heart rate values than the other groups. This indicates that individuals who do not smoke have better cardiorespiratory capacity. Negative effects of smoking on exercise tolerance and maximal oxygen uptake (VO₂ max) are frequently reported in the literature. The aerobic capacity and endurance performance of smokers have been found to be significantly lower [17]. Additionally, smoking reduces the heart rate reserve by limiting the heart rate response during exercise [18]. Epidemiological studies have shown that smoking is inversely related to aerobic capacity [19].

In our study, no statistically significant differences were observed between the groups in terms of the HRV parameters. However, the mean heart rate, RMSSD, and SDNN values remained at the lower limit of the normal range in all the groups. Notably, the heart rate was higher, and the RMSSD/SDNN values were slightly lower in e-cigarette users. The higher heart rate and lower RMSSD/SDNN tendency observed in e-cigarette users suggest that sympathetic activity may be relatively dominant and that the cardiovascular system may be in an early activated state [20]. The negative trend of the PNS indices indicates that parasympathetic activity is not predominant in all groups, and that autonomic balance may have shifted toward the sympathetic side. These findings reveal that HRV has not yet been significantly impaired in young and short-term users but that cigarette and electronic cigarette use may affect the cardiovascular stress response and autonomic balance. In addition, in our study, the differences between the groups in HRV parameters were statistically or clinically minor. This suggests that cigarette and e-cigarette use may have had a limited effect on resting autonomic function in this sample. Long-term follow-up studies are required to determine whether these tendencies lead to permanent cardiac effects. Studies have shown that heavy cigarette smoking reduces cardiac vagal modulation and disrupts autonomic control [21]. Chronic smokers experience a decrease in HRV parameters and smoking cessation improves HRV [22]. It has been reported that changes in HRV were observed in mouse models with acute exposure to e-cigarettes, and that even short-term exposure can affect heart rate and HRV [23]. However, the effects of e-cigarette use on autonomic parameters have not yet been clarified in human studies, and a pilot study found no significant differences in resting HRV values after smoking and vaping [24]. In addition, it has been shown that in e-cigarette users, the orientation of the cardiac autonomic balance shifts toward sympathetic dominance and increases oxidative stress [20]. Although the literature does not show consistent sex-specific effects of smoking on pulmonary function, the predominance of female participants in our sample may have contributed to the observed spirometric and autonomic profiles. Therefore, the observed patterns may partly reflect sex-related physiological differences in addition to the effects of cigarette and e-cigarette use.

In our study, dyspnea scores measured during exercise were lower in e-cigarette users than in non-smokers. This may indicate perceptual differences, user adaptation, or sensory differences in measurements rather than a true physiological advantage. Repeated exposure to airway irritants can lead to sensory adaptation and desensitization of vagal airway afferent pathways, which may blunt the perception of respiratory symptoms over time [25]. Nicotine also has the potential to modulate the central processing of respiratory sensations, thus altering the subjective experience of breathlessness [26]. Additionally, reporting bias may have contributed to this, as users may have normalized or underreported respiratory symptoms.

After the incremental shuttle walk test, the recovery heart rate values at the 1st, 3rd, and 5th minutes remained above 100 beats/min across the three groups, suggesting a relatively delayed autonomic recovery response. Interestingly, traditional cigarette smokers exhibited lower post-test heart rate values than non-smokers and e-cigarette users. When interpreted together with the lower percentage of maximal heart rate and shorter walking distances, this finding may reflect a blunted chronotropic response to exercise rather than more efficient cardiovascular adaptation, suggesting impaired autonomic and cardiovascular responsiveness in this group of patients. Moreover, heart rate response and coronary heart disease are highly correlated [27]. Delayed heart rate recovery after exercise is considered a predictive indicator of coronary heart disease [28]. Failure of the heart rate to decrease rapidly after exercise is considered an indication that parasympathetic reactivation is delayed and sympathetic dominance persists [29]. Tobacco use adversely affects heart rate recovery by increasing sympathetic activity and suppressing vagal tone [30]. Similarly, it has been shown that the use of electronic cigarettes disrupts heart rate regulation owing to their nicotine content and alters autonomic balance by increasing sympathetic activity [31]. Moreover, slow recovery of heart rate after exercise is associated with low physical fitness [32]. These findings are consistent with the results of our study and suggest that both tobacco and electronic cigarette use may adversely affect cardiac autonomic response after exercise. The effect size analysis revealed moderate between-group effects among nonsmokers, traditional cigarette smokers, and e-cigarette users for exercise-related cardiovascular responses, particularly for the percentage of maximal heart rate and post-test heart rate, indicating that differences in autonomic and cardiovascular stress responses became more pronounced under exercise conditions than at rest. A moderate effect size was also observed for functional exercise performance, as reflected by the percentage of predicted walking distance, suggesting meaningful differences in exercise capacity among the three groups.

This study provides important insights into the comparative effects of traditional cigarette smoking and e-cigarette use on respiratory function, autonomic regulation, and exercise capacity among young adults. Although the cross-sectional design limits the ability to establish cause-and-effect relationships, the comprehensive evaluation conducted within the same population, including spirometry, heart rate variability, and functional exercise tests, and the use of objective physiological measurements instead of subjective assessments are the strengths of the study. However, this study had certain limitations. The relatively small sample size and the fact that the sample, particularly the non-smoking group, was predominantly composed of women, may have affected lung function and autonomic responses due to known sex-related physiological differences. In this context, longitudinal studies with larger sample sizes and balanced sex distributions are expected to better elucidate the effects of cigarette and e-cigarette use on the cardiopulmonary system. Future studies should consider incorporating correlation analyses between HRV indices and exercise performance measures to better elucidate the autonomic–functional relationships in smokers.

## References

[1] Polosa R et al (2019) The effect of e-cigarette aerosol emissions on respiratory health: a narrative review. Expert Rev Respir Med 13(9):899–915 31375047 10.1080/17476348.2019.1649146

[2] Caponnetto P et al (2013) Electronic cigarette: a possible substitute for cigarette dependence. Monaldi Arch Chest Dis 79(1) 10.4081/monaldi.2013.104 23741941

[3] Biener L, Hargraves JL (2015) A longitudinal study of electronic cigarette use among a population-based sample of adult smokers: association with smoking cessation and motivation to quit. Nicotine Tob Res 17(2):127–133 25301815 10.1093/ntr/ntu200 PMC4375383

[4] Gotts JE et al (2019) What Are Respiratory Eff e-cigarettes? BMJ 366

[5] Tran L, Tran P, Tran L (2020) A cross-sectional analysis of electronic cigarette use in US adults by asthma status. Clin Respir J 14(10):991–997 32592339 10.1111/crj.13231

[6] Srivastava R, Blackstone EH, Lauer MS (2000) Association of smoking with abnormal exercise heart rate responses and long-term prognosis in a healthy, population-based cohort. Am J Med 109(1):20–26 10936474 10.1016/s0002-9343(00)00441-1

[7] Bodin F et al (2017) The association of cigarette smoking with High-Frequency heart rate variability: an ecological momentary assessment study. Psychosom Med 79(9):1045–1050 28731984 10.1097/PSY.0000000000000507 PMC5675783

[8] Hayano J et al (1990) Short-and long-term effects of cigarette smoking on heart rate variability. Am J Cardiol 65(1):84–88 2294686 10.1016/0002-9149(90)90030-5

[9] Culver BH et al (2017) Recommendations for a standardized pulmonary function report. An official American thoracic society technical statement. Am J Respir Crit Care Med 196(11):1463–1472 29192835 10.1164/rccm.201710-1981ST

[10] Probst VS et al (2012) Reference values for the incremental shuttle walking test. Respir Med 106(2):243–248 21865021 10.1016/j.rmed.2011.07.023

[11] Esco MR, Flatt AA (2014) Ultra-short-term heart rate variability indexes at rest and post-exercise in athletes: evaluating the agreement with accepted recommendations. J Sports Sci Med 13(3):535 25177179 PMC4126289

[12] Schaffarczyk M et al (2022) Validity of the polar H10 sensor for heart rate variability analysis during resting state and incremental exercise in recreational men and women. Sens (Basel) 22(17):6536 10.3390/s22176536 PMC9459793 36081005

[13] Santos-de-Araújo AD et al (2023) Inter- and intrarater reliability of short-term measurement of heart rate variability on rest in chronic obstructive pulmonary disease (COPD). Heart Lung: J Cardiopulm Acute Care 62:64–71 10.1016/j.hrtlng.2023.06.004 37327614

[14] Shaffer F, Ginsberg JP (2017) An overview of heart rate variability metrics and norms. Front Public Health 5:258 29034226 10.3389/fpubh.2017.00258 PMC5624990

[15] Oelsner EC et al (2020) Lung function decline in former smokers and low-intensity current smokers: a secondary data analysis of the NHLBI pooled cohorts study. Lancet Respir Med 8(1):34–44 31606435 10.1016/S2213-2600(19)30276-0 PMC7261004

[16] Wills TA et al (2021) E-cigarette use and respiratory disorders: an integrative review of converging evidence from epidemiological and laboratory studies. Eur Respir J 57(1):1901815 10.1183/13993003.01815-2019 PMC7817920 33154031

[17] Lee CL, Chang WD (2013) The effects of cigarette smoking on aerobic and anaerobic capacity and heart rate variability among female university students. Int J Womens Health 5:667–679 24204174 10.2147/IJWH.S49220 PMC3804543

[18] Chen CL et al (2015) Immediate effects of smoking on cardiorespiratory responses during dynamic exercise: arm Vs. Leg ergometry. Front Physiol 6:376 26696905 10.3389/fphys.2015.00376 PMC4674552

[19] Ho C-C et al (2022) Associations between cigarette smoking status and health-related physical fitness performance in male Taiwanese adults. Front Public Health 10:880572 36062134 10.3389/fpubh.2022.880572 PMC9433563

[20] Moheimani RS et al (2017) Increased cardiac sympathetic activity and oxidative stress in habitual electronic cigarette users: implications for cardiovascular risk. JAMA Cardiol 2(3):278–284 28146259 10.1001/jamacardio.2016.5303 PMC5626008

[21] Barutcu I et al (2005) Cigarette smoking and heart rate variability: dynamic influence of parasympathetic and sympathetic maneuvers. Ann Noninvasive Electrocardiol 10(3):324–329 16029383 10.1111/j.1542-474X.2005.00636.x PMC6932108

[22] Harte CB, Meston CM (2014) Effects of smoking cessation on heart rate variability among long-term male smokers. Int J Behav Med 21(2):302–309 23397454 10.1007/s12529-013-9295-0

[23] Castellanos JA et al (2025) Electronic cigarettes alter cardiac rhythm and heart rate variability hyperacutely in mice. Toxicol Appl Pharmacol 495:117174 39608730 10.1016/j.taap.2024.117174

[24] Shahin K, West SL, Brenner IK (2022) Acute cardiovascular effects of vaping compared to cigarette smoking in young adults. McGill J Med 20(1)

[25] Mazzone SB, Undem BJ (2016) Vagal afferent innervation of the airways in health and disease. Physiol Rev 96(3):975–1024 27279650 10.1152/physrev.00039.2015 PMC4982036

[26] Davenport PW, Vovk A (2009) Cortical and subcortical central neural pathways in respiratory sensations. Respir Physiol Neurobiol 167(1):72–86 18977463 10.1016/j.resp.2008.10.001

[27] Simula S et al (2014) Heart rate variability associates with asymptomatic coronary atherosclerosis. Clin Auton Res 24(1):31–37 24343834 10.1007/s10286-013-0220-z

[28] Akyüz A et al (2014) Heart rate recovery May predict the presence of coronary artery disease. Anadolu Kardiyol Derg 14(4):351–356 24818624 10.5152/akd.2014.4824

[29] Erat M et al (2016) Evaluation of heart rate recovery index in heavy smokers. Anatol J Cardiol 16(9):667–672 27488749 10.5152/AnatolJCardiol.2015.6500 PMC5331350

[30] Papathanasiou G et al (2013) Effects of smoking on heart rate at rest and during exercise, and on heart rate recovery, in young adults. Hellenic J Cardiol 54(3):168–177 23685653

[31] Garcia PD, Gornbein JA, Middlekauff HR (2020) Cardiovascular autonomic effects of electronic cigarette use: a systematic review. Clin Auton Res 30(6):507–519 32219640 10.1007/s10286-020-00683-4 PMC7704447

[32] Arastoo S et al (2020) Acute and chronic sympathomimetic effects of e-cigarette and tobacco cigarette smoking: role of nicotine and non-nicotine constituents. Am J Physiol Heart Circ Physiol 319(2):H262–h270 32559135 10.1152/ajpheart.00192.2020 PMC7473924

